# Palmitoylethanolamide Reduces Neuropsychiatric Behaviors by Restoring Cortical Electrophysiological Activity in a Mouse Model of Mild Traumatic Brain Injury

**DOI:** 10.3389/fphar.2017.00095

**Published:** 2017-03-06

**Authors:** Francesca Guida, Serena Boccella, Monica Iannotta, Danilo De Gregorio, Catia Giordano, Carmela Belardo, Rosaria Romano, Enza Palazzo, Maria A. Scafuro, Nicola Serra, Vito de Novellis, Francesco Rossi, Sabatino Maione, Livio Luongo

**Affiliations:** ^1^Department of Experimental Medicine, Section of Pharmacology “L. Donatelli”, Università degli Studi della Campania “Luigi Vanvitelli” (Ex SUN)Naples, Italy; ^2^Endocannabinoid Research Group, Institute of Biomolecular Chemistry, Consiglio Nazionale delle RicerchePozzuoli, Italy; ^3^Department of Anesthesiology, Surgery and Emergency, Università degli Studi della Campania “Luigi Vanvitelli” (Ex SUN)Naples, Italy; ^4^Department of Radiology, Università degli Studi della Campania “Luigi Vanvitelli” (Ex SUN)Naples, Italy; ^5^Young Against Pain (YAP) Italian Group, NaplesItaly

**Keywords:** traumatic brain injury, Palmitoylethanolamide, medial prefrontal cortex, mice depression, aggressive behavior, *in vivo* electrophysiology

## Abstract

Traumatic brain injury (TBI) represents a major public health problem, which is associated with neurological dysfunction. In severe or moderate cases of TBI, in addition to its high mortality rate, subjects may encounter diverse behavioral dysfunctions. Previous reports suggest that an association between TBI and chronic pain syndromes tends to be more common in patients with mild forms of brain injury. Despite causing minimal brain damage, mild TBI (mTBI) often leads to persistent psychologically debilitating symptoms, which can include anxiety, various forms of memory and learning deficits, and depression. At present, no effective treatment options are available for these symptoms, and little is known about the complex cellular activity affecting neuronal activity that occurs in response to TBI during its late phase. Here, we used a mouse model to investigate the effect of Palmitoylethanolamide (PEA) on both the sensorial and neuropsychiatric dysfunctions associated with mTBI through behavioral, electrophysiological, and biomolecular approaches. Fourteen-day mTBI mice developed anxious, aggressive, and reckless behavior, whilst depressive-like behavior and impaired social interactions were observed from the 60th day onward. Altered behavior was associated with changes in interleukin 1 beta (IL-1β) expression levels and neuronal firing activity in the medial prefrontal cortex. Compared with vehicle, PEA restored the behavioral phenotype and partially normalized the biochemical and functional changes occurring at the supraspinal level. In conclusion, our findings reveal some of the supraspinal modifications responsible for the behavioral alterations associated with mTBI and suggest PEA as a pharmacological tool to ameliorate neurological dysfunction induced by the trauma.

## Introduction

Traumatic brain injury (TBI) represents a major public health problem, which is associated with neurological dysfunction. It may pertain to injuries with severities ranging from moderate to high (Saatman et al., 2008), and may be accompanied by several neurological dysfunctions, inflammatory processes, and cell death (Arciniegas, 2011). TBI is divided into two phases: primary injury and the consequent secondary reaction. It is believed that primary cell death due to the injury cannot really be prevented, whereas the secondary neuropsychiatric changes could potentially be managed by therapeutic intervention because they are driven by several pathogenic parameters (Arciniegas et al., 2005). In the past, it has been demonstrated, in an animal model of mild TBI (mTBI), that the induction of oxidative and nitrosative damage leads to cerebrovascular inflammation (Abdul-Muneer et al., 2013). In fact, several animal models of TBI have been developed (Shultz et al., 2016). Moreover, neuroinflammatory and pro-apoptotic molecules are involved in the early phase of mTBI (Zetterberg et al., 2013). Interestingly, it has also been reported that peripheral immune cells, such as mast cells and T-cells are implicated in this process (Kelso and Gendelman, 2014; Corrigan et al., 2016). Despite recent advances in the knowledge of the mechanisms involved in mTBI, no treatments, except for palliative care, are currently available (Loane and Faden, 2010). It is currently believed that the secondary neuropsychiatric changes that occur as a consequence of TBI are linked to plastic rearrangements in several brain areas including the hippocampal and medial prefrontal cortex (mPFC) circuitry (Schwarzbold et al., 2008). The mPFC is also thought to play a key role in chronic pain-related negative affective states (i.e., anxiety, depression, and cognitive impairments), which are often present as comorbidities accompanying damages to the central or peripheral nervous system (CNS or PNS, respectively), such as neuropathy or trauma. Among the neurotransmitters and neuromodulators involved in the central sequelae of TBI, endocannabinoids (eCBs) have previously been shown to be involved in the pathogenesis of CNS-related disorders, and more recently, in their underlying mechanisms as well (Shohami et al., 2011; Mann et al., 2016). In particular, a recent study indicates that preventing the degradation of endogenous cannabinoid ligands after mild TBI attenuates neuroinflammation and improves the recovery of neurobehavioral function during the first 7 days post-TBI induction (Mayeux et al., 2016). In addition to classical eCBs, endocannabinoid-related N-acylethanolamines, such as Palmitoylethanolamide (PEA) have been shown to participate in TBI-mediated dysfunctions (Ahmad et al., 2012; Cuzzocrea et al., 2012). PEA is an endogenous lipid compound belonging to the fatty acid ethanolamide (FAE) family, which is derived from the reaction between ethanolamine and palmitic acid. PEA is highly expressed in the mammalian brain and exerts considerable pharmacological effects when supplied exogenously (Lo Verme et al., 2005a,b), as a drug, or when its endogenous levels are enhanced through the inhibition of its catabolism (Solorzano et al., 2009). In particular, PEA has been shown to be involved in neuroprotective mechanisms that are activated under several pathological states, including chronic pain (Luongo et al., 2013; D’Agostino et al., 2015; Di Cesare Mannelli et al., 2015; Guida et al., 2015) and other CNS-related disorders associated with neuroinflammation (Skaper et al., 2013).

In the present study, we aimed to characterize the behavioral and electrophysiological phenotype of 60-day mild-TBI mice and to investigate the effect of chronic PEA administration on the associated sensorial and neuropsychiatric dysfunctions. We evaluated (i) chronic pain symptoms (allodynia and hyperalgesia) and affective-cognitive impairments including anxiety/depression, aggressive, recklessness-like behaviors and reduced sociability at 14 and 60 days post-trauma; (ii) the bio-molecular and electrophysiological changes occurring in the m-PFC; and (iii) the effects of repeated PEA administration on behavioral and functional changes in TBI mice.

## Materials and Methods

### Animals

Male C57BL/6 mice (Charles River, Italy) weighing 18–20 g were housed three per cage under controlled illumination (12 h light/dark cycle; light on 6:00 A.M.) and standard environmental conditions (ambient temperature 20–22°C, humidity 55–60%) for at least 1 week before the commencement of experiments. Mice chow and tap water were available *ad libitum*. The experimental procedures were approved by the Animal Ethics Committee of Università della Campania “L. Vanvitelli”, Naples. Animal care was in compliance with Italian (D.L. 116/92) and European Commission (O.J. of E.C. L358/1 18/12/86) regulations on the protection of laboratory animals. All efforts were made to reduce both animal numbers and suffering during the experiments.

### Mild mTBI

Experimental mTBI was performed using a weight-drop device developed in our laboratory. Mice were anesthetized with intraperitoneal injection of Avertin (250 mg/kg) before being subjected to mTBI. After a midline longitudinal incision, the skull was exposed to locate the area of impact and placed under a metal tube device where the opening was positioned directly over the animal’s head. The injury was induced by dropping a cylindrical metal weight (50 g), through a vertical metal guide tube from a height of 20 cm. The point of impact was between the anterior coronal suture (bregma) and posterior coronal suture (lambda). Immediately following injury, the skin was closed with surgical wound clips and mice were placed back in their cages to allow for recovery from the anesthesia and mTBI. Sham mice were submitted to the same procedure as described for mTBI, but without release of the weight.

### Drugs

Ultra micronized (0.8–6.0 μm) Palmitoylethanolamide (μm-PEA) was kindly provided by EPITECH Group SpA, Saccolongo (PD).

### Behavioral Testing

**FIGURE 1:**
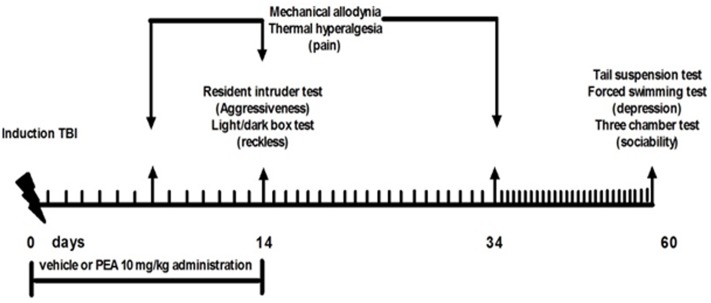
**Timeline of the experimental procedure of mild Traumatic Brain Injury (mTBI) induction and related behavioral characterization in presence of vehicle or Palmitoylethanolamide (PEA) treatment**.

Behavioral tests were performed from day 14 to 60 from mTBI induction. At the end of each set of experiments mice were sacrificed for further evaluations. The behavioral tests were scheduled in order to avoid carry-over effects from prior testing experience. The application of different pain stimuli -mechanical or thermal- was performed in separate groups of animals in order to avoid interferences in the nociceptive response. Timeline of mTBI induction and related behavioral characterization is given in the **Figure 1**.

### Mechanical Allodynia

Mechanical allodynia was evaluated at 7, 14, and 34 days after mTBI or sham surgery by the Dynamic Plantar Aesthesiometer (Ugo Basile, Varese, Italy). Mice were allowed to move freely in 1 of the 2 compartments of the enclosure positioned on the metal mesh surface. Mice were adapted to the testing environment for 30 min before any measurement was taken. After that, the mechanical stimulus was delivered to the plantar surface of the hindpaw of the mouse from below the floor of the test chamber by an automated testing device. A steel rod (2 mm) was pushed with electronical ascending force (0–30 g in 10 s). When the animal withdrawn its hindpaw, the mechanical stimulus was automatically withdrawn and the force recorded to the nearest 0.1 g. Nociceptive responses for mechanical sensitivity were expressed as mechanical withdrawal thresholds (MWT) in grams. Each mouse served as its own control, the responses being measured both before and after surgical procedures. MWT were quantified by an observer blind to the treatment.

### Thermal Hyperalgesia

Thermal hyperalgesia was evaluated at 7, 14, and 34 days after mTBI or sham surgery by the Plantar Test Apparatus (Ugo Basile, Varese, Italy). On the day of the experiment each mouse was placed in a plastic cage (22 cm × 17 cm × 14 cm; length × width × height) with a glass floor. After 30 min habituation period, the plantar surface of the hind paw was exposed to a beam of radiant heat through the glass floor. The radiant heat source consists of an infrared bulb (Osram halogen-bellaphot bulb; 8 V, 50 W). A photoelectric cell detected light reflected from the paw and turned off the lamp when paw movement interrupted the reflected light. Data were expressed as thermal withdrawal latency (TWL) in seconds and TWL was automatically displayed to the nearest 0.1 s; the cut-off time was 20 s in order to prevent tissue damage. Each mouse served as its own control, the responses being measured both before and after surgical procedures. TWL were quantified by an observer blind to the treatment.

### Light-Dark Box

At 14 days after mTBI or sham surgery, mice were tested to measure reckless behavior using ligh/dark box test. The light–dark box (600 mm × 300 mm × 300 mm; length × width × height) apparatus consisted into two equally sized compartments: the dark compartment (black perspex) was covered, whereas the light compartment (white perspex) was open and brightly lit from above (∼150 lux). Access between compartments was allowed through a partition door (70 mm × 70 mm). At the beginning of the session, mice were placed in the light compartment and were free to explore for 10 min. The time spent in in each compartment, latency of first entry into the dark compartment and number of entries into each compartment were recorded.

### Resident-Intruder Test

At 14 days after mTBI or sham surgery, mice were tested for aggressive behavior using a resident intruder test. Mice were individually housed for 1 week in Plexiglas cages to establish a home territory and to increase the aggression of the resident experimental mice. To begin, food containers were removed and an intruder mouse of the same gender was placed in a resident home cage and resident–intruder interactions were analyzed for 10 min. The aggressive behavior of resident socially isolated mice was characterized by an initial pattern of exploratory activity around the intruder, which was followed by rearing and tail rattle, accompanied in few seconds by wrestling and/or a violent biting attack. The number of these attacks and latency to the first attack during 10 min observation period was recorded.

### Three Chambers Sociability Test

At 60 days after mTBI or sham surgery, mice were tested for social interaction using a three-chambered social interaction apparatus. A plexi-glass three-chambered box was custom-built as follows: doorways in the two dividing walls had sliding covers to control access to the outer-side chambers. The test consisted of two consecutive stages of 5 and 10 min each. During the 5-min first stage of habituation the mouse was allowed to freely explore the three chambers of the apparatus, detecting at this stage any innate side preference. After that the mouse was gently encouraged into the central chamber and confined there briefly by closing the side chamber doors. During the following 10-min stage sessions, a custom-made stainless-steel barred cup (6.5 cm × 15 cm) was placed upside down in one of the side chambers. A never-before-met intruder, previously habituated, was placed into an upside down identical cup in the other chamber. The time spent sniffing each upside-down cup, the time spent in each chamber and the number of entries into each chamber were recorded.

### Forced Swimming Test (FST)

The forced swimming test (FST) and tail suspension test (TST) are used to assess and quantify the extent to which a mouse model displays a depression-like activity. TST and FST were evaluated at 60 after mTBI or sham surgery, respectively.

#### Forced Swimming Test

Mice were placed in a large cylinder (30 cm × 45 cm) filled with water at a temperature of 27°C, for a 6-min period. The duration of immobility was monitored during the last 4 min of the 6-min test. Immobility period was defined as the time spent by the animal floating in the water without struggling and making only movements necessary to keep its head above the water. Immediately afterward, the trial mice were placed under a heating lamp to dry.

#### Tail Suspension Test (TST)

Mice were individually suspended by the tail on a horizontal bar (50 cm from floor) using adhesive tape placed approximately 4 cm from the tip of the tail. The duration of immobility, recorded in seconds, was monitored during the last 4 min of the 6-min test by a time recorder. Immobility time was defined as the absence of escape-oriented behavior. Mice were considered to be immobile when they did not show any body movement, hung passively and completely motionless.

### *In vivo* Electrophysiology

#### Single Extracellular Neurons Recordings

Mice for electrophysiological recordings were anesthetized with pentobarbital (50 mg/kg, i.p.) and placed in a stereotaxic device (David Kopf Instruments, Tujunga, CA, USA). Body temperature was maintained at 37°C with a temperature-controlled heating pad. In all surgical preparations, the scalp was incised and holes were drilled in the skull overlying the site of recording, mPFC (AP: +1.54–1.78 mm from bregma, L: 0.3–0.5 from midline and V: –1.5–3 mm below dura) (**Figure 4A**) and the site of stimulation, BLA (AP: –0.5–2.06 mm from bregma, L: 2.8–3.0 from midline at anteroposterior angle of 30°, and V: –4.2–5 below dura) according to the coordinates from the atlas of Franklin and Paxinos (1997) (**Figure 4B**). Anesthesia was maintained with a constant continuous infusion of propofol (5–10 mg/kg/h, i.v.). A bipolar concentric electrode (NEX-100; Rhodes Medical Instruments Inc., Summerland, CA, USA) connected to A320 stimulator (World Precision Instruments England) was lowered into the caudal region of the BLA. After lowering of the stimulating electrode into the BLA, a glass-insulated tungsten filament electrode (3–5 MX) (FHC Frederick Haer & Co., Bowdoinham, ME, USA) was stereotaxically lowered into the prelimbic cortex (PLC) in mPFC. The recorded signals were amplified and displayed on a digital storage oscilloscope to ensure that the unit under study was unambiguously discriminated throughout the experiment. Signals were processed by an interface CED 1401 (Cambridge Electronic Design Ltd., UK) and analyzed through Spike2 software (CED, version 4) to create peristimulus rate histograms (PSTHs) online and to store and analyze digital records of single-unit activity off-line. Configuration, shape, and height of the recorded action potentials were monitored and recorded continuously. This study only included neurons with a regular spiking pattern and a spontaneous firing rate between 0.1 and 3.82 Hz that were classified as pyramidal neurons in rodents (Jung et al., 1998; Tierney et al., 2004; Floresco and Tse, 2007). Once a neuron was single out, the position of the microelectrode was adjusted to maximize the spike amplitude relative to background noise and electrical stimuli into the BLA were delivered.

#### Characterization of BLA-Evoked Responses and Stimulation Protocol

We observed that BLA stimulation could evoke two distinct types of firing changes: inhibition or excitation in separate populations of mPFC neurons (Ishikawa and Nakamura, 2003). Neurons responsive to BLA stimulation were stimulated with 100–200 pulses to determine the cell type. Specifically, a cell was considered to be excited by BLA stimulation if it displayed a fast-onset increase of firing and hereafter referred as “BLA→PLC(+)” neurons (Floresco and Tse, 2007). This group of neurons showed a cluster of spikes typically showing a Poisson distribution and displayed an increased probability of spike firing after BLA stimulation (200 μA) (**Figure 4C**). From the PSTHs, we measured the duration of excitation (in seconds) as the period of the increased firing activity which exceeds the average baseline value +2SDs, the frequency of the evoked excitatory responses and the onset of excitation, which was considered as the time from the application of the stimulus artifact to the first evoked spike exceeding the average baseline value +2SDs. These criteria were used as an index of changes in the excitatory influence that BLA inputs exert over mPFC neuron.

A second group of neurons displaying a complete cessation of spontaneous firing after BLA stimulation they are classified as “BLA→mPFC(–)” neurons (Floresco and Tse, 2007) (**Figure 4D**). Once a neuron, that was inhibited by BLA stimulation, was isolated and characterized, a single-pulse stimulation was delivered at 0.5 Hz using an initial stimulation current of 200 μA. We typically used 100–250 sweeps to generated on-line PSTHs. We measure the duration of inhibition (in seconds), the period when the spontaneous firing was completely suppressed, and the onset of inhibition (in milliseconds), the time interval between the stimulus application and last spike before a complete cessation of neuronal activity, after BLA stimulation as defined by Ishikawa and Nakamura (2003). By using these parameters, we could have a reliable index of changes in the inhibitory influences that BLA inputs exert over the mPFC neuron firing (Ishikawa and Nakamura, 2003; Laviolette and Grace, 2006; Floresco and Tse, 2007).

Mechanical stimuli were applied to the hind paw (contralateral to the mPFC) by Von Frey filaments with bending force of 97.8 mN (noxious stimulation) for 2 s (Simone et al., 2008) The mechanical stimulus evoked inhibitory or excitatory response in separate populations of mPFC neurons. The same parameters of the inhibitory and excitatory responses were measured and considered as an index of mPFC neuron firing response to mechanical noxious stimuli (Giordano et al., 2012). Neurons that displayed no change in firing in response to electrical or mechanical stimulation, were not considered for recording.

Moreover, the extracellular action potentials’ (EAP) amplitude, indicating synaptic current was used for the evaluation of the efficacy of synaptic transmission (Fagni et al., 1987). Data analysis was performed with Spike2 software version 7.0.

### Biochemical Analysis

#### Protein Extraction and Western Blot Analysis

For protein extraction, the PL-IL cortex was minced into small pieces with a blender, then suspended in lysis buffer [HEPES 25 mM; EDTA 5 mM; SDS 1%; Triton X-100 1%; PMSF 1 mM; MgCl2 5 mM; Protease Inhibitor Cocktail (Roche, Mannheim, Germany); Phospahatase Inhibitor Cocktail (Roche, Mannheim, Germany)] and cleared by centrifugation (10 min at 10,000 ×*g* at 4°C). Protein concentration was determined using the method described by Bradford (1976). Each sample was loaded (30 μg), electrophoresed in a 8 or 15% SDS-polyacrylamide gel and electroblotted onto a polyvinylidene difluoride (PVDF) membrane (EMD Millipore Corp., Billerica, MA, USA). The membrane was blocked in 5% milk, 1X Tris-buffered saline and 0,05% Tween-20. Primary antibodies to detect IL-1β (1:500, Santa Cruz Biotechnology, Santa Cruz, CA, USA), IL-10 (1:500, Santa Cruz Biotechnology, Santa Cruz, CA, USA) Cox-2 (1:500, Santa Cruz Biotechnology, Santa Cruz, CA, USA), I-NOS (1:1000, Abcam, Cambridge, UK) were used according to the manufacturer’s instruction. Immunoreactive signals were detected with a horseradish peroxidase-conjugated secondary antibody and reacted with an ECL system (Amersham Pharmacia, Uppsala, Sweden). Protein levels were normalized with respect to the signal obtained with anti-beta-actin monoclonal antibodies (Sigma Chemical Co. 1:1000 dilution). The semi-quantitative analysis of protein levels was carried out by the ChemiDoc-It 5000, using VisionWorks Life Science Image Acquisition and Analysis software (UVP, Upland, CA, USA). Bradford M.M. A rapid and sensitive method for the quantization of microgram quantities of protein utilizing the principle of protein-dye binding.

### Statistical Analysis

Data were represented as mean ± SEM. The behavioral data (*n* = 8–9) were analyzed using One-way Anova, followed by Tukey *post hoc*. Electrophysiological data (*n* = 3–4) were analyzed using one-way ANOVA, followed by Holm–Sidack *post hoc* comparisons or *t*-test. Biomolecular data (*n* = 3) were analyzed by One-way Anova, followed by Student–Neuman–Keuls test.

## Results

### PEA Reduces mTBI-Induced Pain Behaviors

**FIGURE 2:**
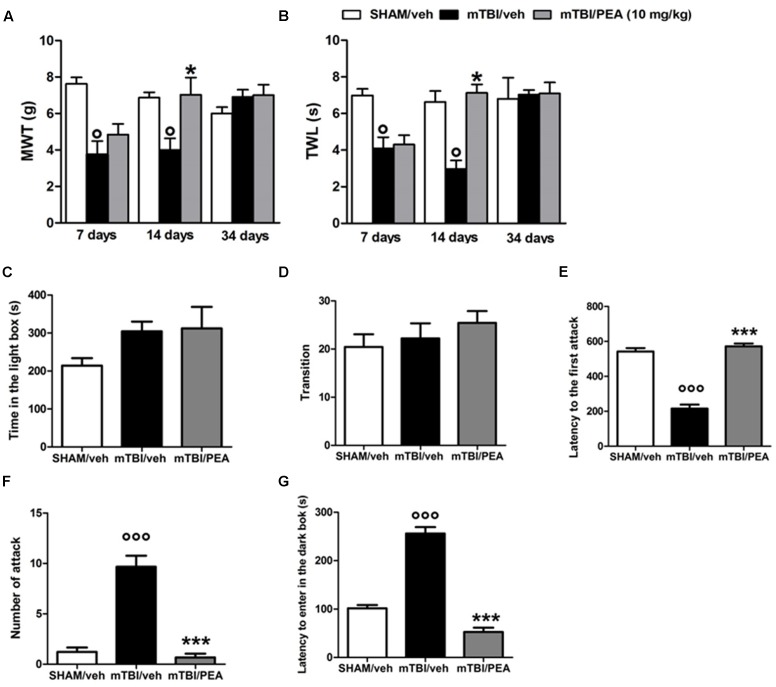
**Effects of repeated administration (14 days) of vehicle (Pluronic acid 5%) or PEA (10 mg/kg, i.p.) on behavioral evaluations in sham and mTBI mice. (A)** Shows mechanical withdrawal thresholds (MWT) measured through dynamic plantar aesthesiometer, **(B)** shows the thermal withdrawal latency (TWL) in the plantar test, **(C–E)** show the latency to enter in the dark box, the time spent in light box and the number of transition in the light/dark box test, respectively. **(F,G)** Show the latency to the first attack and the number of attack in the resident intruder test, respectively. Data are represented as mean ± SEM of eight mice per group. ° and ^∗^ indicate significant differences compared to sham/vehicle or TBI/vehicle, respectively. *P* < 0.05 was considered statistically significant. One-way ANOVA, followed by Tukey *post hoc*.

A significant decrease of MWT and TWL was observed in vehicle-treated mTBI mice at 7 and 14 days after trauma induction (MWT: 3.7 g ± 0.7, *F*_(2,21)_ = 14.26, *P* = 0.0001, and 4 g ± 0.6, respectively; TWL: 4.1 s ± 0.6, *F*_(2,21)_ = 11.69, *P* = 0.0004, and 3.0 s ± 0.5, respectively) compared to the sham group (MWT: 7.6 g ± 0.3; MWT and 6.9 g ± 0.3, respectively; TWL: 7 s ± 0.4 and 6.6 s ± 0.6, respectively) (**Figures 2A,B**). No difference in pain threshold was observed between right and left paw (data not shown). Moreover, a complete physiological re-establishment of normal pain response was observed 34 days after trauma induction (MWT: 6.9 g ± 0.4, *F*_(2,21)_ = 1.112, *P* = 0.3476; TWL:7.0 s ± 0.2, *F*_(2,21)_ = 0.05148, *P* = 0.9499) compared to the sham mice (MWT: 6.0 g ± 0.3; TWL: 6.8 s ± 1.1) (**Figure 2A,B**). PEA repeated treatment (10 mg/kg, i.p., 14 days) significantly reduced both the mechanical allodynia and thermal hyperalgesia in mTBI mice at 14 days (MWT: 7.0 g ± 0.9, *F*_(2,21)_ = 7.014, *P* = 0.0046; TWL: 7.1 s ± 0.5, *F*_(2,21)_ = 16.62, *P* < 0.0001) as compared with vehicle (MWT: 4.0 g ± 0.6; TWL: 3.0 s ± 0.5) (**Figures 2A,B**). No difference was observed between right and left paw with PEA treatment (data not shown). The vehicle or PEA administration in sham mice did not change the pain response compared to naive or sham/vehicle mice (data not shown).

### PEA Reduces mTBI-Induced Recklessness-Like Behaviors

Compared to the controls (214.2 s ± 19.5 and 20.44 ± 2.6), mTBI mice did not show any significant change in the time spent in the illuminated compartment of the light/dark box (315.8 s ± 29.6, *F*_(2,12)_ = 2, *P* = 0.71), as well as, in the number of transitions in the two compartments(22.22 s ± 3.1; *F*_(2,24)_ = 0.85; *P* = 0.53) at 14 days post trauma (**Figures 2C,D**). However, mTBI condition caused a significantly increase in the latency to enter in the dark box (256.2 s ± 13.2), that we identify as recklessness-like behaviors (**Figure 2E**), as compared to the control animals (101.6 s ± 6.7). The treatment with PEA (10 mg/kg, i.p., 14 days) significantly decreased this effect (52.5 s ± 9, *F*_(2,24)_ = 112,9 *P* < 0.01) as compared with vehicle (256.2 s ± 13.2), while did not affect the total time spent in the light compartment and the number of transitions mediated by mTBI (**Figures 2C–E**). Sham mice treated whit vehicle or PEA did not show any change in time spent in the illuminated compartment of the light/dark box, in the number of transition in the two compartments or in the latency to enter the dark compartment compared to naive or sham/vehicle mice (data not shown).

### PEA Reduces mTBI-Induced Aggressive Behavior

Vehicle-mTBI mice showed a shorter latency to the first attack (215.9 s ± 22.6) compared to sham animals (542.1 s ± 19.7) 14 days post trauma (**Figure 2F**). Additionally, compared to the controls (1.2 ± 0.4), the number of attacks was dramatically increased (9.7 ± 1.2) (**Figure 2G**). PEA administration (10 mg/kg, i.p., 14 days) increased the latency to the first attack (571.7 s ± 16.2; *F*_(2,24)_ = 100.5; *P* < 0.001) and reduced the number of attacks (0.7 ± 1.4, *F*_(2,24)_ = 49.25; *P* < 0.001) of mTBI mice, as compared with vehicle (215.9 s ± 22.6 and 9.7 ± 1.2, respectively) (**Figures 2F,G**). Sham mice treated whit vehicle or PEA did not show any change in the latency to the first attack or number of attacks compared to naive or sham/vehicle mice (data not shown).

### PEA Reduces the mTBI-Induced Depressive-Like Behavior

**FIGURE 3:**
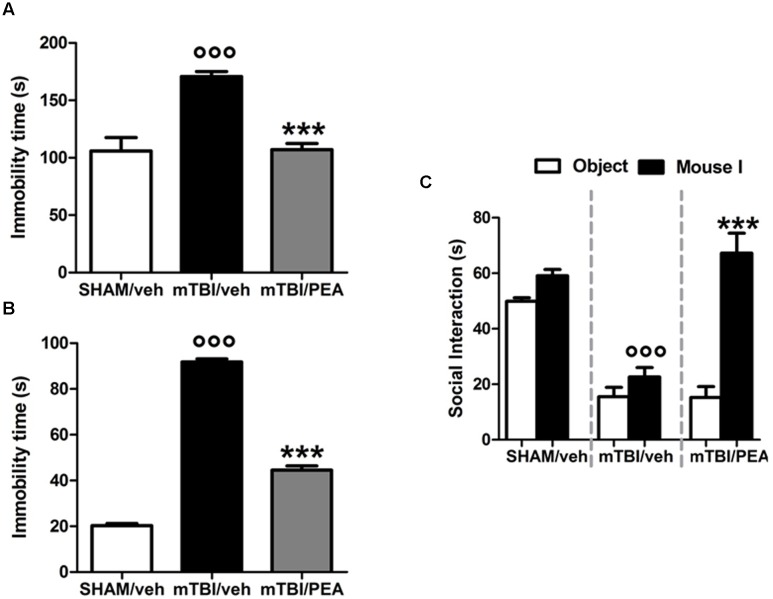
**Effects of repeated administration (14 days) of vehicle (Pluronic acid 5%) or PEA (10 mg/kg, i.p.) on behavioral evaluations in sham and mTBI mice. (A,B)** Show the duration of immobility in the tail suspension test and in the forced swimming test, respectively. **(C)** Shows the duration of the time spent with an object or mouse in the three chambers sociability apparatus. Data are expressed in seconds and represented as mean ± SEM of 8–9 mice per group. ° and ^∗^ indicate significant differences compared to sham/vehicle or TBI/vehicle, respectively. *P* < 0.05 was considered statistically significant. One-way ANOVA, followed by Tukey *post hoc*.

mTBI mice showed an increased immobility time, measured as the lack of escape-oriented activity (s) (91.7 s ± 1.3) compared to the sham mice (20.2 s ± 1.0) in TST 60 days post trauma (**Figure 3A**). Similarly, we found an enhanced duration of immobility, measured as the time spent by the animal floating in the water, in the FST (170.8 s ± 4.3) compared to the controls (105.9 s ± 11.7) (**Figure 3B**). PEA treatment (10 mg/kg, i.p., 14 days) significantly reduced the immobility in mTBI condition in both paradigms (tail suspension: 44.5 s ± 1.8, *F*_(2,21)_ = 636.4, *P* < 0.0001; forced swimming: 107.1 s ± 5.4, *F*_(2,21)_ = 22.35; *P* < 0.001) compared to the vehicle (tail suspension: 91.7 ± 1.3 s; forced swimming: 170.8 s ± 4.3) (**Figures 3A,B**). Sham mice treated whit vehicle or PEA did not show any change in the duration of immobility compared to naive or sham/vehicle mice (data not shown).

### PEA Reduces the mTBI-Induced Impaired Social Behavior

Analysis of the social preference revealed an impairment of social interaction which occurred 60 days post trauma. Indeed, mTBI mice showed reduced sociability level, spending a lower time in interacting with the objector mouse during the recorded time (15.5 s ± 3.4), compared to sham animals (49.9 s ± 1.3) (**Figure 3C**). No difference in the time spent in each chamber or in the number of transitions between the chambers was observed in mTBI mice treated with vehicle compared to the sham mice treated with vehicle (not shown). The treatment with PEA (10 mg/kg, i.p., 14 days) significantly improved the social behavior in mTBI mice (67.2 s ± 7.1; *F*_(2,21)_ = 41.81, *P* < 0.001) compared to the with vehicle (15.5 s ± 3.4). Sham mice treated whit vehicle or PEA did not show any change in sociability, the time spent in the two chambers or in the number of transitions between the chambers compared to naive or sham/vehicle mice (data not shown).

### Characterization of Electrical and Mechanical Stimulation-Evoked Responses of mPFC(+) Neurons in SHAM and TBI Mice 14 and 60 Days after Trauma

**FIGURE 4:**
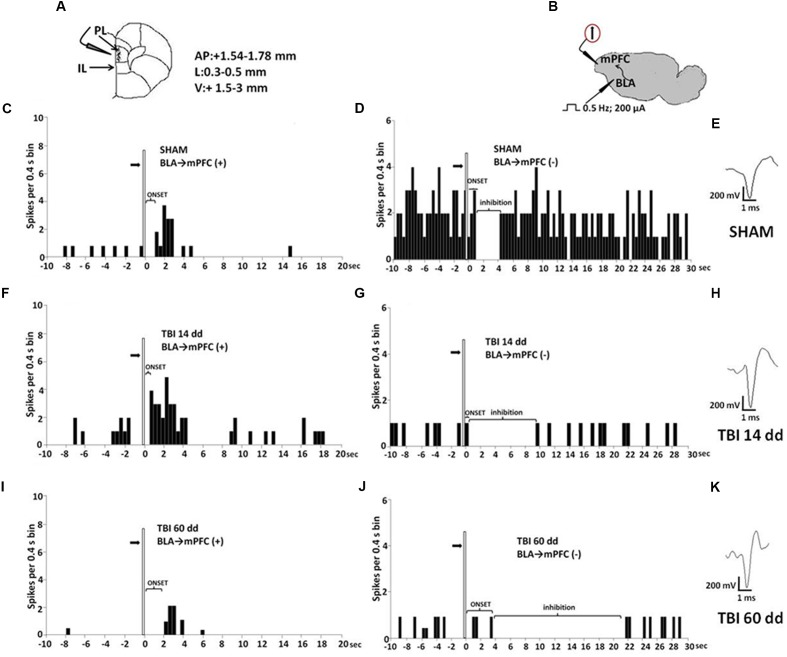
**Schematic illustration of the mPFC recording sites which are indicated by filled circles**. Number values in mm indicate distance from bregma **(A)**. Representative schematic illustration showing the location of the recording electrode in cortex and of the stimulation electrode in the BLA. Number values refer to stimulation parameters **(B)**. BLA electrical stimulation evokes excitatory [BLA → mPFC(+)] or inhibitory [BLA → PLC(–)] responses in different mPFC neuron populations. **(C,D,F,G,I,J)** Show peristimulus time histograms (PSTHs) of SHAM **(C,D)**, TBI 14 days **(F,G)** and TBI 60 days mice **(I,J)** receiving subcutaneous vehicle injection. PSTHs show responses of a single neuron to a pulse (200 μA intensity, black arrows) delivered at in the BLA at 0.5-Hz stimulation (vertical bar). Representative average waveforms of SHAM **(E)**, TBI 14 days **(H)**, and TBI 60 days mice **(K)**. Horizontal bar = 1 ms; vertical bar = 200 mV.

Single-unit extracellular recordings in anesthetized mice were performed from individual neurons in the mPFC (**Figure 4A**). The slow spontaneous firing rate of about 0.3–3.82 ± 0.4 spikes/s from recorded neurons was consistent with presumed pyramidal cells (Pérez-Jaranay and Vives, 1991; Floresco and Tse, 2007), rather than fast-spiking interneurons, the latter having a higher baseline firing rate (>10 Hz) and narrower spike waveform (<300 μs) (Constantinidis and Goldman-Rakic, 2002; Laviolette and Grace, 2006). Firing rates were measured as the average rate in spikes/s.

We investigated the mPFC neurons with ongoing activity that responded with excitation [BLA→PLC(+)] and with inhibition [BLA→mPFC(–)] to electrical and mechanical stimulation (Gabbott et al., 2006; **Figure 4**). We first isolated mPFC neurons and thereafter stimulated the BLA at 0.5 Hz using a stimulation current of 200 μA (**Figure 4B**). Electrical stimulation outside of the BLA did not induce any significant change in mPFC pyramidal neuron activity. The neurons were first identified by electrical stimulation, and subsequently stimulated with mechanical noxius stimuli on the hind paw using von Frey filaments (with bending force of 97.8 mN for 2 s; Simone et al., 2008) for all the duration of the experiment (each single neuron was recorded for at least 20 min) (Luongo et al., 2012).

**FIGURE 5:**
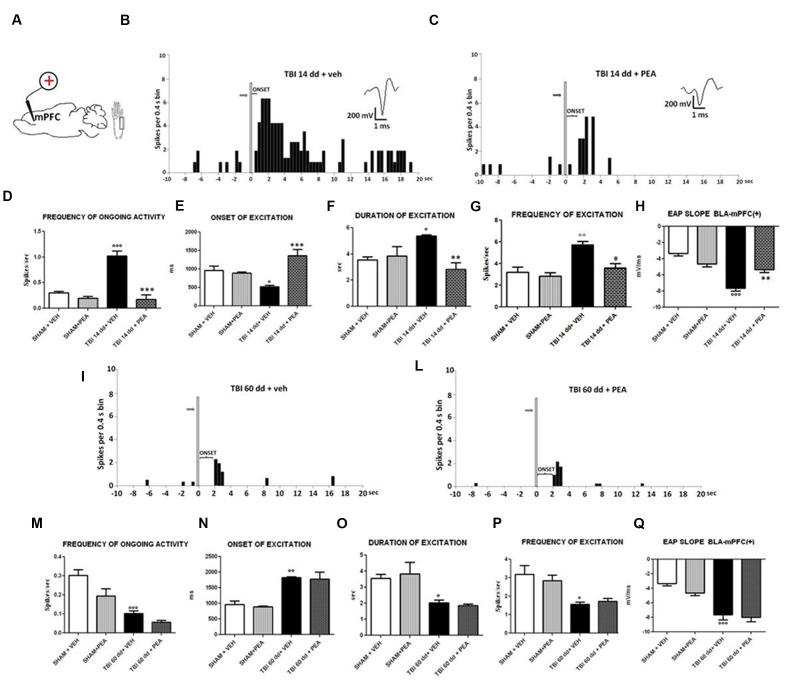
**The figure shows different parameters of mechanical stimulus-evoked excitation in SHAM and TBI mice 14 and 60 days after trauma, treated with vehicle or PEA (10 mg/kg, i.p.) for 14 days. (A)** Shows schematic illustration of mechanical stimulation applied to the hind paw (contralateral to the mPFC) which evokes excitatory responses in the prelimbic cortex (PLC). **(B,C,I,J)** Show representative PSTHs of a BLA→mPFC (+) neuron of SHAM and TBI 14 and 60 dd mice treated for 14 days with vehicle or PEA. Vertical bars indicate the mechanical stimuli which evoke excitation (hind paw stimulation by von Frey filaments with bending force of 97.8 mN, gray arrows). (**D–G,K–O**) Show the mean ± standard error (SEM) of the mean of spontaneous activity, of the frequency, of the duration and of the onset of excitation in different groups of mice. **(H,O)** Show relative EAP slopes recorded in different groups of mice. °Indicates statistically significant difference versus SHAM/veh mice; ^∗^indicates statistically significant difference versus TBI 14 dd/veh or TBI 60dd/mice. *p* < 0.05 has been considered as value of significance. The double symbol indicates *p* < 0.01 and the triple symbol indicates *p* < 0.001. One-way ANOVA, followed by Holm–Sidack *post hoc* comparisons or *t*-test.

BLA→PLC(+) neurons had a spontaneous firing rate of 0.30 ± 0.031 spikes/s in SHAM/veh group (**Figure 4C**). The frequency, the duration and the onset of excitation were 3.18 ± 0.48 spikes/s, 3.56 ± 0.23 s and 960.1 ± 122 ms, respectively, in SHAM/veh group (**Figures 5E–G**) (*n* = 9 neurons recorded from 3 to 4 mice per group). TBI mice 14 days after induction of trauma (TBI 14 dd/veh) showed an increase in the spontaneous firing rate (1.020 ± 0.10 spikes/s; *F*_(3,8)_ = 31.24, *p* < 0.001) (*n* = 9 neurons recorded from 3 to 4 mice per group) (**Figure 5B**). Moreover, an increase in the frequency of stimulation-evoked excitation of BLA→mPFC (+) neurons (5.73 ± 0.33 spikes/s*; F*_(3,8)_ = 10.92, *p* < 0.01) and in the duration of excitation (5.36 ± 0.12 s; *F*_(3,8)_ = 5.34, *p* < 0.05) was observed, while the onset was reduced (524.7 ± 32.38 ms; *F*_(3,8)_ = 10.66, *p* < 0.05) (**Figures 5E–G**). TBI mice 60 days after induction of trauma (TBI 60 dd/veh) showed a further impairment in electrophysiological parameters. In particular, the spontaneous activity was significantly reduced (0.10 ± 0.01 spikes/s) with respect to both TBI 14 dd/veh group (*p* < 0.001) and to SHAM/veh group (*F*_(3,8)_ = 16.77, *p* < 0.001) (*n* = 9 neurons recorded from 3 to 4 mice per group) (**Figure 5K**). With a similar fashion, the frequency and the duration of excitation were decreased (1.55 ± 0.12 spikes/s and 2.03 ± 0.16 s, respectively) with respect to both TBI 14 dd/veh group (*p* < 0.01) and to SHAM/veh group (*F*_(3,8)_ = 7.05, *p* < 0.05 and *F*_(3,8)_ = 6.86, *p* < 0.05, respectively) (**Figures 5M,N**). Whereas, the onset was significantly increased (1824 ± 34.19 ms) as compared to both TBI 14 dd/veh group (*p* < 0.05) and to SHAM/veh group (*F*_(3,8)_ = 25.14, *p* < 0.01). We quantified the slope of the initial negative-going EAP response, which reflects the early activation of the mPFC. In SHAM mice the slope of the EAP was –3.33 ± 0.33 mV/ms and it significantly increased 14 days (–7.66 ± 0.33 mV/ms, *F*_(3,8)_ = 18.47, *p* < 0.001) and 60 days (–7,66 ± 0.66 mV/ms, *F*_(3,8)_ = 20.85, *p* < 0.001) after trauma (**Figures 5H,O**, respectively).

### Characterization of Electrical and Mechanical Stimulation-Evoked Responses of mPFC(–) Neurons in SHAM and TBI Mice 14 and 60 Days after Trauma

**FIGURE 6:**
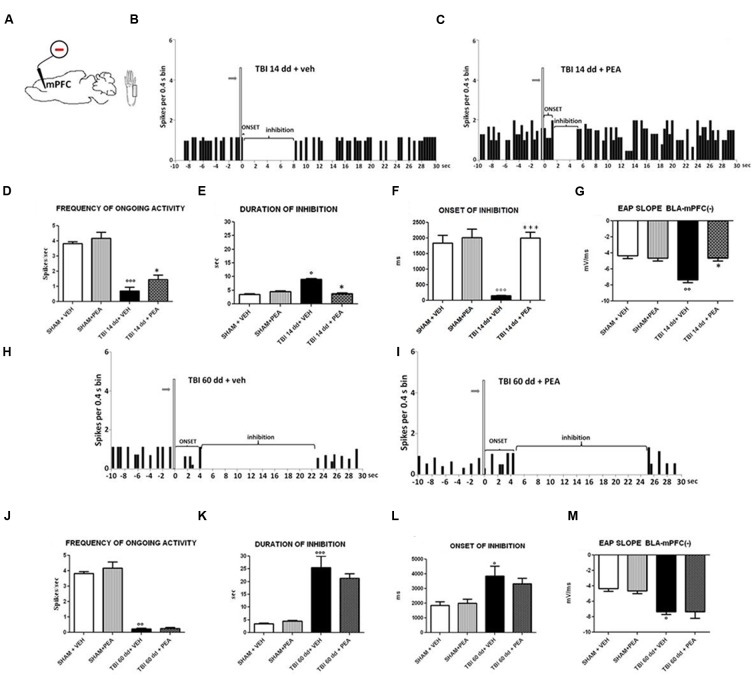
**The figure shows different parameters of mechanical stimulus-evoked excitation in SHAM and TBI mice 14 and 60 days after trauma, treated for 14 days with vehicle or PEA (10 mg/kg, i.p.). (A)** shows schematic illustration of mechanical stimulation applied to the hind paw (contralateral to the mPFC) who evokes inhibiting responses in the prelimbic cortex. **(B,C,H,I)** Show representative PSTHs of a BLA→mPFC (–) neuron of SHAM and TBI mice 14 and 60 days after trauma, treated for 14 days with vehicle or PEA. Vertical bars indicate the mechanical stimuli which evoke inhibition (hind paw stimulation by von Frey filaments with bending force of 97.8 mN, gray arrows). **(D–F,J–L)** Show the mean ± standard error of the spontaneous activity, of duration and of onset of inhibition in different groups of mice. **(G,M)** show relative EAP slopes recorded in different group of mice. ° indicates statistically significant difference versus SHAM/veh mice; ^∗^ indicates statistically significant difference versus TBI 14 dd/veh or TBI 60 dd/veh mice. *p* < 0.05 has been considered as value of significance. The double symbol indicates *p* < 0.01 and the triple symbol indicates *p* < 0.001. One-way ANOVA, followed by Holm–Sidack *post hoc* comparisons or *t*-test.

BLA→PLC(–) neurons had a spontaneous firing rate of 3.82 ± 0.12 spikes/s in SHAM/veh group (**Figure 6D**). The duration and the onset of inhibition were 3.54 ± 0.29 s and 1844 ± 247.6 ms, respectively (**Figures 6E,F**) (*n* = 9 neurons recorded from 3 to 4 mice per group). TBI mice 14 days after induction of trauma (TBI 14 dd/veh) showed an increase in the duration of inhibition (8.97 ± 0.38 s; *F*_(3,8)_ = 97.95, *p* < 0.05) and a decrease in the onset (153.6 ± 11.78 ms; *F*_(3,8)_ = 19.04, *p* < 0.001) and in the frequency of ongoing activity (0.71 ± 0.23 spikes/s; *F*_(3,8)_ = 36.81, *p* < 0.0001) of BLA→mPFC (–) neurons (*n* = 9 neurons recorded from three mice per group) (**Figures 6D–F**). TBI mice, 60 days after induction of trauma (TBI 60 dd/veh), showed severe changes in electrophysiological parameters. In fact, the duration (1824 ± 34.19 ms; *F*_(3,8)_ = 48.83, *p* < 0.001) and the onset of inhibition (25.50 ± 4.50 ms; *F*_(3,8)_ = 5.34, *p* < 0.05) were significantly increased while the frequency of ongoing activity (0.23 ± 0.04 spikes/s; *F*_(3,8)_ = 100.9, *p* < 0.01) was significantly reduced as compared to SHAM/veh group (**Figures 6J–L**). The TBI also induced a significant increase of EAP slope values at 14 days (–7.66 ± 0.33 mV/ms; *F*_(3,8)_ = 17.58, *p* < 0.01) and 60 days (7,66 ± 0.66 mV/ms; *F*_(3,8)_ = 9.7, *p* < 0.05), as compared to the controls (4.33 ± 0.33 mV/ms) (**Figures 6G,M**).

### Effect of PEA on BLA→PLC(+) Neurons in TBI Mice 14 and 60 Days after Trauma

Repeated treatments with PEA (10 mg/kg) did not affect neither spontaneous nor stimulus-evoked activity of BLA→mPFC(+) neurons in SHAM mice (*n* = 9 neurons recorded from 3 to 4 mice) as compared to vehicle-treated group (**Figure 5**). On the contrary, TBI 14 dd mice treated with PEA (10 mg/kg i.p.) showed a normalization in the frequency of ongoing activity (0.17 ± 0.09 spikes/s, *F*_(3,8)_ = 31.24, *p* < 0.001), in the duration of evoked frequency (2.84 ± 0.5 s, *F*_(3,8)_ = 5.34, *p* < 0.001), in the frequency of excitation (3.58 ± 042 spikes/sec; *F*_(3,8)_ = 10.92, *p* < 0.05) and in the EAP slope (–5.33 ± 0.33 mV/ms, *F*_(3,8)_ = 29.58, *p* < 0.01) (**Figures 5D–H**). At the same time, a significant increase in the onset of excitation (1366 ± 166.5 ms, *F*_(3,8)_ = 10.66, *p* < 0.001) was observed, as compared to TBI 14 dd/vehicle (**Figure 5E**). The treatment with PEA (10 mg/kg) did not affect the electrophysiological parameters in TBI 60 days after trauma (**Figure 5**).

### Effect of PEA on BLA→PLC(–) Neurons in TBI Mice 14 and 60 Days after Trauma

Repeated treatments with PEA (10 mg/kg) did not affect neither spontaneous nor stimulus-evoked activity in BLA→mPFC(–) neurons in SHAM mice (*n* = 9 neurons recorded from 3 to 4 mice). Treatment with PEA (10 mg/kg i.p.) normalized the duration of inhibition (3.78 ± 0.24 s, *F*_(3,8)_ = 67.95, *p* < 0.05) and the EAP slope (–4.66 ± 0.33 mV/ms, *F*_(3,8)_ = 17.58, *p* < 0.05) (**Figures 6E,G**).

Instead, the firing rate (1.44 ± 0.29 spikes/sec, *F*_(3,8)_ = 36.81, *p* < 0.05) and the onset of inhibition (1999 ± 187.5 ms, *F*_(3,8)_ = 19.04, *p* < 0.001) resulted increased in TBI 14 dd mice as compared to TBI 14 dd/vehicle (**Figures 6D,F**). The treatment with PEA (10 mg/kg) did not affect the electrophysiological parameters previously analyzed in TBI 60 days after trauma (**Figure 6**).

### PEA Normalizes the mTBI-Induced Increased IL-1β Levels

Western blot analysis revealed a significant increase of the pro-inflammatory cytokine IL-1β protein levels in the PL-IL of TBI/vehicle-treated mice as compared to SHAM/vehicle animals 14 days after injury. Instead protein levels of IL-10, iNOS, and COX-2 did not change in TBI mice. Treatment with PEA (10 mg/kg/day, i.p.) decreased the protein levels of IL-1β in TBI mice (*F*_(2,6)_ = 24.75, *p* < 0.05), without affecting IL-10 (*F*_(2,6)_ = 2.10), I-NOS (*F*_(2,6)_ = 0.71), and COX-2 (*F*_(2,6)_ = 0.56) protein levels (see **Table 1** and Supplementary Figure S1). For all proteins examined, naïve and SHAM animals showed no differences in the expression levels (data not shown).

**Table 1:** The protein levels (mean ± SEM) of the proteins under analysis measured by western blot are reported.

Protein (CTX)	SHAM/vehicle	TBI/vehicle	TBI/PEA ultra
IL-ip/p-actin	100.0 ± 10.0	150.0 ± 4.0°	78.0 ± 7.0^∗^
IL-10/p-actin	100.0 ± 22.5	84.0 ± 7.5	55.0 ± 13.4
COX-2/p-actin	100.0 ± 13.3	100.0 ± 9.8	114.0 ± 8.8
I-NOS/p-actin	100.0 ± 28.6	112.0 ± 29	67.0 ± 25

*The analysis of protein levels was carried out by the ChemiDoc-It 5000, using VisionWorks Life Science Image Acquisition and Analysis software (UVP, Upland, CA, USA). The measured protein levels were normalized with respect to β-actin (housekeeping gene) and protein expression values were expressed as arbitrary units ± SEM. ° vs Sham/vehicle and ^∗^*p* < 0.05 vs. TBI/vehicle, as analyzed by One-way ANOVA, followed by Student–Neuman–Keuls test*.

## Discussion

Despite early brain damage, mild TBI is commonly associated with a late manifestation of psychologically debilitating symptoms and cognitive impairments, for which adequate treatment is currently unavailable. In the present study, we have characterized the behavioral phenotype associated with the late phase of TBI (up to 60 days post-trauma), as well as have investigated possible supraspinal changes, i.e., cytokine profile and electrophysiological neuronal activity. Our results show that mTBI induces a characteristic dual behavioral phenotype (aggressive/depressive), possibly by regulating biochemical and electrophysiological processes in the mPFC. As has been previously reported in humans (DSM V), mTBI mice developed aggressive and recklessness-like behaviors in an early phase; 2 weeks after trauma induction. At this stage, they also displayed abnormal pain perception, described as mechanical allodynia. Chronic pain is a common comorbidity of TBI (Ofek and Defrin, 2007; Nampiaparampil, 2008), and nociceptive sensitization of areas that are far from the injury site has previously been observed (Feliciano et al., 2014). Though the mechanism by which TBI may affect the peripheral nociceptive threshold is unknown, recent data suggest that brain damage may induce an increase in proinflammatory molecules that then leak to the periphery, contributing to the development of pain (Feliciano et al., 2014; Rowe et al., 2016). In the current study, the recklessness-like behavior and abnormal pain perception were self-limited after 2 and 3 weeks, respectively. Interestingly, aggressiveness, which was still present 60 days after trauma, was accompanied by depressive-like behavior, determined by absence of escape-oriented activity and increased immobility. Furthermore, TBI animals showed social interaction impairments, which are also frequently observed in patients and are thought to reflect underlying cognitive impairments caused by the trauma (Byom and Turkstra, 2016).

Our data also revealed that mTBI deeply affects cortical neuronal activity. In fact, a similar behavioral profile was correlated with the biphasic firing activity of the pyramidal neurons in the mPFC, which is considered a key substrate regulating negative affective states, including anxiety and depression (Vialou et al., 2014; Apps and Strata, 2015).

Specifically, (BLA)-mPFC(–) inhibitory and BLA-mPFC(+) excitatory neurons, which concurrently respond to hind-paw pressoceptive stimuli, showed phenotypic changes following brain injury, suggesting that the mPFC may undergo profound reorganization in response to the trauma. Additionally, 14 days after trauma, cells that had an excitatory response to stimulation, showed an increase in both their spontaneous and evoked firing activity, whereas those of inhibitory cells appeared reduced. However, at 60 days of mTBI, we observed a pathological cortical hypofunctionality, characterized by an overall depressed pattern of neuronal activity. These findings suggest that brain trauma may implicate an alteration of the balance between excitatory and inhibitory transmission in the BLA-mPFC circuit, and consequently impair the broad complexity of tasks attributed to PFC activity. Previous studies have shown that an increased inflammatory response associated with microglial activation, peripheral immune cell infiltration, and cytokine release is elicited within a few days following a traumatic episode (Hall et al., 2010; Woodcock and Morganti-Kossmann, 2013; Gao et al., 2016). Interestingly, we found increased levels of interleukin 1 beta (IL-1β) at cortical levels, 14 days post-trauma, whereas no differences in the expression levels of classical pro-inflammatory mediators, including iNOS and COX2, were observed. The augmented levels of supraspinal IL-1β and the subsequent IL-1 receptor activation is a central mechanism in several pathological conditions (Giordano et al., 2012; Lampa et al., 2012). In agreement with its pro-inflammatory profile, previous studies also proved that IL-1β has a direct influence on cortical synaptic transmission and plasticity, which may increase the risk of cognitive decline and dysfunction (Tong et al., 2012; del Rey et al., 2013). Moreover, direct IL-1β-mediated plasticity has been shown in patients with multiple sclerosis (Mori et al., 2014; Nisticò et al., 2014). Thus, based on the present results, we can hypothesize that IL-1β might also be involved in the remodeling and/or increased activity of the mPFC pyramidal neurons as has previously been shown for chronic pain conditions (Giordano et al., 2012).

In this study, we also confirm the beneficial effects of PEA, a commercially available medical supplement, on the psychiatric outcomes of TBI. The anti-inflammatory/neuroprotective effects of PEA have been clearly shown in several pathological conditions, including TBI (Ahmad et al., 2012; Cordaro et al., 2016). However, here we show the effectiveness of PEA in reducing mTBI-induced late-onset (60 days post-trauma) psychiatric dysfunctions as well as its effect on the modulation of neuronal activity. Specifically, PEA reverted recklessness-like behaviors and abnormal pain perception (observed at 14 days) as well as normalizing the emotional/affective impairments (depression and impaired sociability), which developed 60 days post-trauma. Importantly and in accordance with the behavioral data, electrophysiological experiments revealed that PEA normalized the firing activity of the pyramidal neurons in the mPFC of 14-day-TBI mice. This confirms the role of this lipid signaling molecule in processes related to pain as well as its involvement in neurological and emotional processes linked to CNS damage (Martinez-Vargas et al., 2013). Surprisingly, while PEA reverted the depression and reduced sociability seen in 60-day TBI-mice, it was not able to modify the observed mPFC electrophysiological changes. This suggests the involvement of other brain areas, including the hippocampus and/or other complex circuitries in the altered neuropsychiatric behavioral profile of mTBI mice. Nevertheless, we cannot exclude the possibility that the rearrangement of cortical activity, detected 60 days after mTBI induction, may have been responsible for other behavioral changes, including cognition, memory and reward, which were not analyzed in the present study.

## Conclusion

Our results demonstrate that TBI causes late sensorial affective/cognitive impairments linked to altered levels of cortical IL-1β, as well as phenotypic changes in the pyramidal neurons of the mPFC. These might represent the effect of a chronic maladaptive process that arises in response to injury-induced damage. Moreover, we showed that chronic PEA treatment partially reduces behavioral dysfunctions by restoring cortical biochemical and electrophysiological processes. Taken together, our results suggest that treatment with PEA could represent a novel approach for the management of neuropsychiatric disorders associated with TBI.

## Author Contributions

FG, SM, LL: designed and wrote the paper. SB, MI, CB, RR, DDG, CG: performed experiments. EP, VdN, FR, MS: contributed materials/analysis tools. NS: revised statistics.

## Conflict of Interest Statement

The authors declare that the research was conducted in the absence of any commercial or financial relationships that could be construed as a potential conflict of interest.
