# Supplementary material for: Palmitoylethanolamide Reduces Neuropsychiatric Behaviors by Restoring Cortical Electrophysiological Activity in a Mouse Model of Mild Traumatic Brain Injury
Source: Front Pharmacol. 2017 Mar 6;8:95. doi: 10.3389/fphar.2017.00095 (PMC5337754; doi:10.3389/fphar.2017.00095)
Supplement: Supplementary file 1 [file Data_Sheet_1.PDF]

Supplementary Material

Palmitoylethanolamide reduces neuropsychiatric behaviors by restoring cortical electrophysiological activity in a mouse model of mild traumatic brain injury

Francesca Guida[1,2#], Serena Boccella[1#], Monica Iannotta[1#], Danilo De Gregorio[1], Catia Giordano[1], Carmela Belardo[1], Rosaria Romano[1] Enza Palazzo[1], Maria Antonietta Scafuro[3], Nicola Serra[4], Vito de Novellis[1], Francesco Rossi[1], Sabatino Maione[1,2]* and Livio Luongo[1,2,5]*

[1]Department of Experimental Medicine, Section of Pharmacology "L. Donatelli", Università degli Studi della Campania "Luigi Vanvitelli" (Ex SUN), 80138 Naples, Italy.

[2]Endocannabinoid Research Group, Institute of Biomolecular Chemistry, Consiglio Nazionale delle Ricerche, Pozzuoli, Italy

[3]Department of Anesthesiology, Surgery and Emergency, Università degli Studi della Campania "Luigi Vanvitelli" (Ex SUN), 80138 Naples, Italy.

[4]Department of Radiology Università degli Studi della Campania "Luigi Vanvitelli" (Ex SUN), 80138 Naples, Italy.

[5]Young Against Pain (YAP) Italian group, Italy.

*** Correspondence:** Dr. Livio Luongo: *livio.luongo@gmail.com*

Figure 1

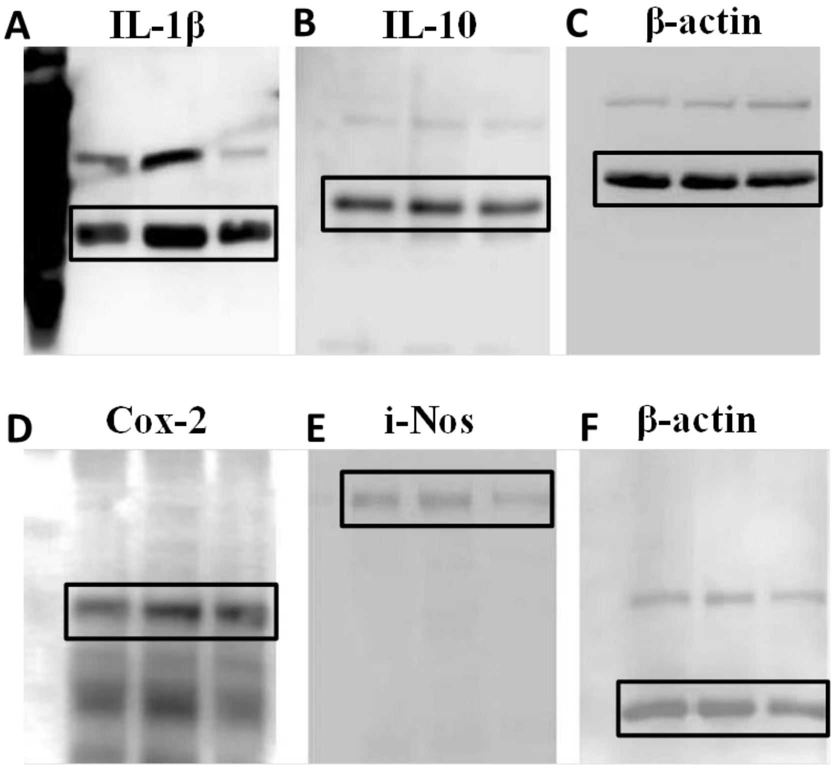

Supplementary Figure 1.

Representative western blot image of IL1β (31kDa) and IL-10 (37 kDa) (A and B) or Cox-2 (70-72 kDa) and i-NOS (130 kDa) (D and E) bands in the cortex of different groups of treatment represented in the table 1. The bands are shown in the box and indicate Sham/Vehicle, TBI/Vehicle and TBI/PEAultra from left to right, respectively. C and F are the representative western blot image of β-actin in the same samples.
